# Glucose Alters *Per2* Rhythmicity Independent of AMPK, Whereas AMPK Inhibitor Compound C Causes Profound Repression of Clock Genes and AgRP in mHypoE-37 Hypothalamic Neurons

**DOI:** 10.1371/journal.pone.0146969

**Published:** 2016-01-19

**Authors:** Johanneke E. Oosterman, Denise D. Belsham

**Affiliations:** 1 Department of Physiology, University of Toronto, Toronto, ON, Canada; 2 Department of Endocrinology and Metabolism, Academic Medical Center, University of Amsterdam, Amsterdam, The Netherlands; 3 Department of Obstetrics and Gynecology, University of Toronto, Toronto, ON, Canada; 4 Department of Medicine, University of Toronto, Toronto, ON, Canada; 5 Division of Cellular and Molecular Biology, Toronto General Hospital Research Institute, University Health Network, Toronto, ON, Canada; University of Cordoba, SPAIN

## Abstract

Specific neurons in the hypothalamus are regulated by peripheral hormones and nutrients to maintain proper metabolic control. It is unclear if nutrients can directly control clock gene expression. We have therefore utilized the immortalized, hypothalamic cell line mHypoE-37, which exhibits robust circadian rhythms of core clock genes. mHypoE-37 neurons were exposed to 0.5 or 5.5 mM glucose, comparable to physiological levels in the brain. *Per2 and Bmal1* mRNAs were assessed every 3 hours over 36 hours. Incubation with 5.5 mM glucose significantly shortened the period and delayed the phase of *Per2* mRNA levels, but had no effect on *Bmal1*. Glucose had no significant effect on phospho-GSK3β, whereas AMPK phosphorylation was altered. Thus, the AMPK inhibitor Compound C was utilized, and mRNA levels of *Per2*, *Bmal1*, Cryptochrome1 *(Cry1)*, agouti-related peptide (*AgRP)*, carnitine palmitoyltransferase 1C (*Cpt1c)*, and O-linked N-acetylglucosamine transferase (*Ogt)* were measured. Remarkably, Compound C dramatically reduced transcript levels of *Per2*, *Bmal1*, *Cry1*, *and AgRP*, but not *Cpt1c* or *Ogt*. Because AMPK was not inhibited at the same time or concentrations as the clock genes, we suggest that the effect of Compound C on gene expression occurs through an AMPK-independent mechanism. The consequences of inhibition of the rhythmic expression of clock genes, and in turn downstream metabolic mediators, such as AgRP, could have detrimental effects on overall metabolic processes. Importantly, the effects of the most commonly used AMPK inhibitor Compound C should be interpreted with caution, considering its role in AMPK-independent repression of specific genes, and especially clock gene rhythm dysregulation.

## Introduction

The endogenous pacemaker, or central clock, is located in the suprachiasmatic nucleus (SCN) in the hypothalamus in mammals. This paired nucleus generates an approximate 24 h rhythm by means of a transcriptional-translational feedback loop [1], which is set to the exact 24 h cycle by light that reaches the SCN via the retinohypothalamic tract (RHT) [2, 3]. The core of this oscillator is formed by the transcription factors circadian locomotor output cycles kaput (CLOCK) and brain and muscle ARNT-like 1 (BMAL1), which drive the expression of three *Period (Per 1–3)* and two *Cryptochrome (Cry 1–2)* genes by binding to their E-box promoter elements. In the cytoplasm, PER and CRY proteins accumulate in a rhythmic manner, then heterodimerize and translocate to the nucleus to suppress their own transcription by interacting with the CLOCK:BMAL1 complex. An additional, a stabilizing loop is formed by the orphan nuclear receptor reverse erythroblastosis virus α (REV-ERBα), which represses, and retinoid-related orphan receptor (ROR), peroxisome proliferator-activated receptor-α (PPAR-α), and PPAR-α coactivator 1-α (PGC1-α), which activate, the transcription of *Bmal1* [4–6]. Post-translational modifications, including phosphorylation and ubiquitination, determine the pace of oscillation [4]. The SCN transmits its signals to the rest of the body through endocrine, behavioral and autonomic pathways [7, 8]. In addition to the central clock in the SCN, peripheral clocks are located in nearly every cell of the body, including non-SCN brain areas [6, 9]. Light does not reach peripheral clocks and therefore they are reset by signals from the SCN, as well as by external signals, including circulating nutrients [10, 11].

Since approximately 8–10% of the transcriptome is under SCN control, including key rate-limiting enzymes, proper functioning of the clock is of great importance for a myriad of physiological processes, including metabolism [12, 13]. The circadian clock and metabolism show an intricate, and bi-directional, relationship. For instance, mutations in either of the two positive regulators of the circadian molecular clock, *Clock* and *Bmal1*, lead to metabolic abnormalities [14, 15], whereas circulating nutrients can affect the molecular clocks, as reviewed in Oosterman et al. [16]. For instance, fatty acids can alter the molecular circadian clockwork, both *in vivo* [17, 18] and *in vitro* [19]. Glucose is a potent entraining factor for the clock. In fibroblasts, glucose has been shown to be an important factor to induce cellular circadian rhythms [20]. Furthermore, glucose can regulate the pace of the peripheral molecular clock through its interplay with phosphorylation and ubiquitination of clock proteins [21, 22].

One of the important functions of the clock is to anticipate the changing environment, including daily fluctuations in circulating glucose and glucose uptake [23–25]. Therefore, it is clear that nutrient sensing and the clock are intimately linked. Nutrient sensors that are able to link the inherent nutrient state to the clock include AMP-activated protein kinase (AMPK) [26] and glycogen synthase kinase 3 beta (GSK3β) [27, 28]. AMPK is a kinase that is activated upon depletion in cellular energy and regulates metabolism and whole-body energy balance [29]. Hypothalamic AMPK regulates energy balance by altering metabolism and food intake in response to nutritional and endocrine food signals [30]. AMPK exerts multiple effects on the clock, including the circadian phosphorylation of CRY1, thereby destabilizing the CRY/PER heterodimer and affecting period length. In synchronized mouse fibroblasts, activation of AMPK reduced the amplitude and increased the period of the circadian rhythm of a luciferase reporter gene driven by the BMAL1-CLOCK promoter in an AMPK-dependent manner [31]. The ubiquitous kinase GSK3β regulates a number of cellular functions, including glucose homeostasis. The kinase activity of GSK3β is circadian and GSK3β has been shown to alter clock period length, although some studies show opposing effects [28, 32, 33]. High concentrations of glucose (30 mM) and insulin (1 nM) have been shown to increase the phosphorylation of GSK3β in a cell model of murine kidney cells [34]. Despite these and other studies, the exact mechanisms through which nutrients can affect the clock, *in vitro* and *in vivo*, remain to be identified.

To study the effect of nutrients on the clock, peripheral cell models have been used, whereas few studies focused on the effects on the hypothalamus. The heterogeneity of the hypothalamus and previous lack of appropriate hypothalamic models has interfered with gaining insight into this relationship. As the hypothalamus is important for homeostatic regulation of feeding and metabolism (reviewed in [35]), we aimed to investigate the effect of varying concentrations of glucose on the rhythmic expression of the core clock genes *Per2* and *Bmal1* in the non-SCN, immortalized murine hypothalamic cell line mHypoE-37 [36], and assessed putative mechanisms through which this occurs. The mHypoE-37 neuronal cells endogenously express key circadian molecular genes [19] and will be used to test the hypothesis that glucose can alter the molecular clock in hypothalamic neurons. Understanding this process will provide insight into the intricate relationship between the circadian clock and metabolism.

## Materials and Methods

### Cell culture techniques

The mHypoE-37 neuronal cell line was previously characterized, and shown that they express the core circadian genes, and that the transcript levels of *Bmal1*, *Per2*, *Rev-erbα*, and *Cry1* endogenously cycle with a circadian period [19]. Cell lines are currently available through CELLutions Biosystems, Inc. (Burlington, ON). mHypoE-37 neurons were grown in DMEM (Sigma-Aldrich, Oakville, Ontario, Canada) containing 5.5 mM glucose, supplemented with 5% FBS (GIBCO, Burlington, Ontario, Canada) and 1% Penicillin-Streptomycin (Gibco, Burlington, Ontario, Canada), as previously described when the cell lines were first generated [36]. Cultures were kept in standard cell culture conditions (37°C, 5% CO2). For the glucose experiments, cells were serum-starved for 12 h in DMEM (D5030, Gibco) lacking FBS, supplemented with 0.5 mM glucose (D-(+)-glucose, Sigma-Aldrich), 4 mM L-glutamine, 1% Penicillin-streptomycin, 3.7 g/L sodium bicarbonate, and 0.11 g/L pyruvate. Following serum starvation, media was aspirated and replaced with DMEM (D5030) containing 5% FBS with either 0.5 mM or 5.5 mM (final concentration) glucose added. RNA was then isolated every 3 hours over a 36 hour period. Serum starvation leads to quiescence of cells [37]. The subsequent replacement with fresh media synchronized the cells, as previously reported [20].

For the experiments with Compound C, cells were serum-starved for 11 h in DMEM (D5030) supplemented with 0.5 mM glucose. Following serum starvation, 0.5 ml of 25 μM (final concentration) Compound C (Dorsomorphin dihydrochloride, Tocris Bioscience, Bristol, UK), dissolved in glucose-free, serum-free DMEM was added to the cells. Control plates received 0.5 ml of glucose-free, serum-free DMEM. Following 1 h pre-treatment, media was aspirated and replaced by DMEM containing either 0.5 mM or 5.5 mM glucose in addition to 12.5 μM (final concentration) Compound C or water. Next, RNA and protein were isolated from plates at indicated time points. Prior to these experiments, increasing doses of Compound C (12.5 μM– 50 μM) were tested for their ability to inhibit AMPK phosphorylation. 12.5 μM Compound C was shown to inhibit AMPK phosphorylation most effectively, whereas 50 μM of Compound C paradoxically increased AMPK phosphorylation. 12.5 μM Compound C had no effect on the viability of the cells (data not shown).

### Quantitative RT-PCR (qRT-PCR)

Total RNA extraction and reverse transcription were performed as previously described [36, 38]. In brief, RNA was isolated using a modified guanidinium thiocyonate method. Genomic DNA was eliminated using the Turbo DNase kit (Ambion, Streetsville, Ontario, Canada), followed by cDNA formation. Single stranded cDNA required for real time quantitative reverse transcriptase PCR (qRT-PCR) was synthesized using the High-Capacity cDNA Reverse Transcription Kit (Applied Biosystems, Streetsville, Ontario, Canada) as per manufacturer’s instructions. cDNA was amplified using Platinum SYBR Green qPCR SuperMix-UDG with ROX (Invitrogen) and gene-specific primers. Primer sequences for *Per2 and Bmal1* [38] and *Histone 3a*, *AgRP* and *Cpt1c* [39] were previously reported. Primer sequence *Ogt*: forward: 5’GGAAGAAGCCAAGGCATGTTAT3’; reverse: 5’AAACACAGCCGAGATTACTCCAG3’. Samples and reagents were loaded in technical triplicates into 384-well plates and were amplified and detected using Applied Biosystems Prism 7000 Sequence Detection System machine (ABI, Streetsville, Ontario, Canada). Results were analyzed using the ABI Sequence Detection System (SDS) version 2.4 software and reported as absolute quantification. Primer concentrations and dose curve were optimized such that the R-squared for the dose curve is 0.95 or better, and that the efficiency is as close to the optimal -3.32 (100% efficiency) as possible. All data were normalized against *Histone 3a* mRNA expression.

### Western blot analysis

Cells were treated as described above and protein was harvested at indicated time points after media change using 1 × cell lysis buffer (Cell Signaling Technology Inc., Danvers, MA, USA) supplemented with 1 mM PMSF, 1% phosphatase inhibitor cocktail 2 and 1% protease inhibitor (Sigma-Aldrich). A total of 25–30 μg of protein was subjected to 8% SDS–PAGE, and transferred onto 0.22 μm PVDF membrane (Bio-Rad, Mississauga, ON, Canada). Membranes were blocked for 1 h at room temperature in Tris-buffered saline with Tween-20 buffer (0.1% TBS-T) and 5% non-fat dry milk followed by an overnight incubation with phospho-specific or total antibodies (1:1000, Cell Signaling Technology Inc.) Blots were then washed in 0.1% TBS-T and incubated with HRP-conjugated secondary antibodies (1:5000, Cell Signaling Technology Inc.) for 1 h at room temperature. Bands were visualized by enhanced chemiluminescence using ECL Select Western blotting detection reagent (GE Healthcare Life Sciences, Pittsburgh, PA, USA) and quantified with densitometry using Image J software (NIH, USA). Obtained values were normalized to their respective total protein levels.

### Statistical analysis

A repeated measures ANOVA was performed to determine statistical differences between the groups at individual time points. Next, a cosinor analysis was used to determine the exact period (i.e. the time that is needed to fulfill a complete cycle), amplitude (i.e. the difference between the peak or trough and the mean value of a cosine curve), and acrophase (i.e. the phase angle of the peak of a cosine curve) for each gene with the statistical software OriginPro: Release 8.5 (OriginLab Corp, Northampton, MA) using the following function: y = mesor + amplitude*cos((2*pi*(x-acrophase))/period). For other experiments, according to the nature of the data, a one-way analysis of variance (ANOVA) or repeated measures ANOVA, followed by post-hoc Tukey’s test, was conducted using IBM SPSS Statistics version 21 (IBM Corp., Armonk, NY, USA) to determine statistical differences between treatment groups. P-values <0.05 were considered significant.

## Results

### 5.5 mM glucose alters the rhythmic expression pattern of Per2

**Fig 1:**
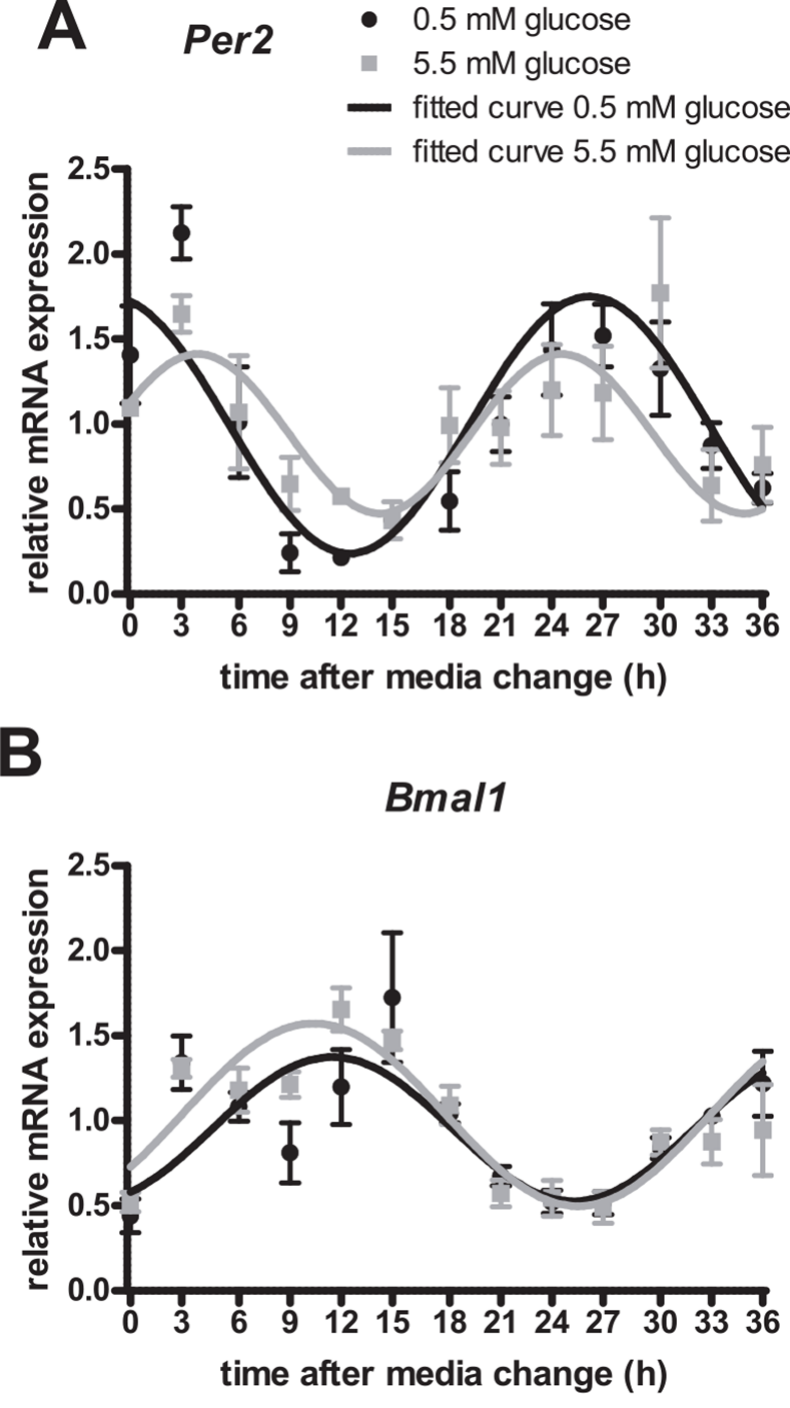
Effects of glucose on the circadian expression profile of *Per2* and *Bmal1*. Following serum starvation, mHypoE-37 neurons were exposed to media containing either 0.5 mM or 5.5 mM glucose. This was considered time point 0. RNA was harvested every 3 h for 36 h and subsequently used for qRT-PCR. All values were normalized to *Histone 3a*. *Ai* and *Bi*: relative mRNA transcript levels of *Per2* and *Bmal1*, respectively. *Per2* and *Bmal1* transcript levels at individual time points were not significantly different between the two glucose concentrations, as determined by repeated measures ANOVA; *Aii*, *Bii*: Transcript levels of the individual groups as shown in *Ai* and *B*i were subjected to cosinor analysis to determine period length, amplitude, and acrophase of each gene. Relative mRNA expression levels of *Per2* and *Bmal1* respectively (similar to *Ai* and *Bi*) are plotted together with the obtained fitted curves from the cosinor analysis. Black circles denote 0.5 mM glucose; grey squares denote 5.5 mM glucose. Black line denotes fitted curve for 0.5 mM glucose, while the grey line denotes fitted curve for 5.5 mM glucose. 5.5 mM glucose significantly (p = 0.02) shortened the period, and caused a phase-delay in the expression pattern of *Per2* (p = 0.02). Values are plotted as mean ± standard error of the mean (SEM) of 4 independent experiments.

mHypoE-37 neurons were serum- and glucose-starved overnight and were replaced with fresh media containing either 0.5 or 5.5 mM glucose. Transcript levels of *Per2* and *Bmal1* were analyzed by qRT-PCR over a 36 h period and were expressed in a rhythmic manner. A cosinor analysis was used to describe and compare the circadian expression pattern between the groups. *Per2* expression differences were found comparing the fitted curves. 5.5 mM glucose significantly (-6.1 h; p = 0.02) shortened the period, and caused a phase-delay in the expression profile of *Per2* (-4.4h h; p = 0.02) (Fig 1A). The amplitude was not significantly different between the 5.5 mM and 0.5 mM group. Expression levels of *Bmal1* did not differ significantly between the cells treated with 0.5 or 5.5 mM glucose, nor did the cosinor analysis reveal significant differences in period, amplitude, or acrophase of *Bmal1* between the groups (Fig 1B).

### Addition of glucose affects phosphorylation levels of AMPK and GSK3β in mHypoE-37 neurons

The nutrient sensors AMPK and GSK3β were studied in order to assess putative mechanisms through which glucose can affect the rhythmic expression of clock genes. Previously we noticed that in cells grown in media without glucose, *Per2* expression showed a high peak at 3 hours after media exchange, coinciding with high levels of phosphorylated AMPK after a 12 h fast in serum-free, glucose-free media, which was absent in cells grown in media containing 5.5 mM glucose (Oosterman JE and Belsham DD, unpublished observations). This led us to hypothesize that activation of AMPK is correlated to *Per2* expression. However, due to the intrinsic need for glucose as fuel for basal metabolism, comparisons with a glucose-free control are not physiologically relevant. In addition, glucose starvation is an important stressor for cells, and therefore the experiment was repeated using 0.5 mM and 5.5 mM glucose.

**Fig 2:**
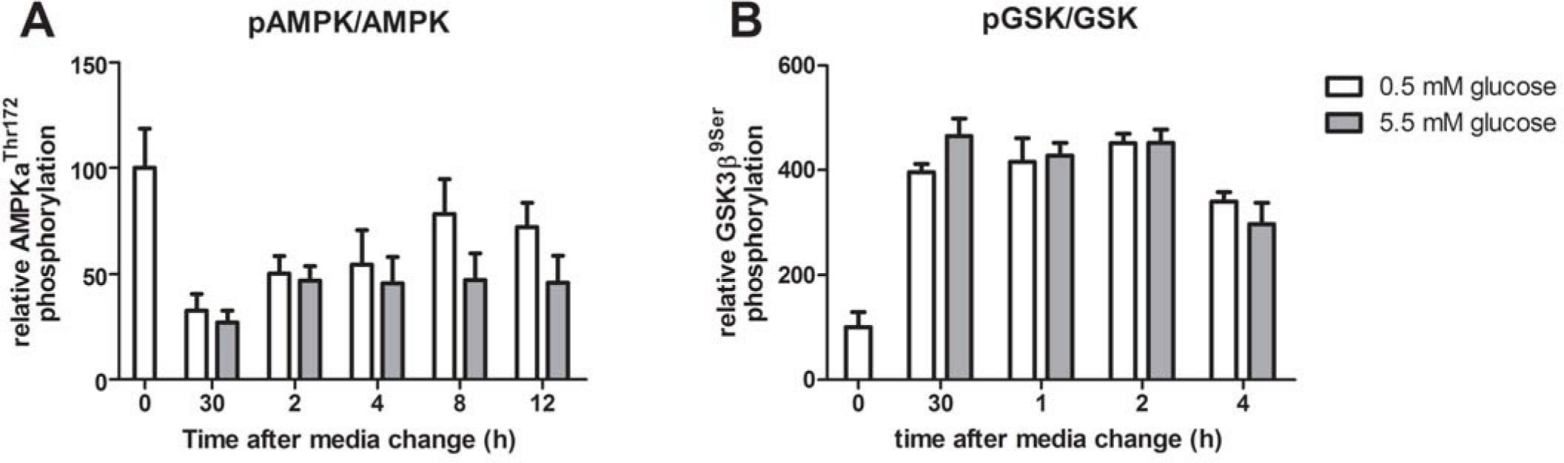
Phosphorylation status of AMPK and GSK3β after media change with either 0.5 mM or 5.5 mM glucose. After an overnight starve in serum-free, 0.5 mM glucose containing media, media was aspirated and fresh media was added containing either 0.5 or 5.5 mM glucose. Protein was harvested at the indicated time points. A) Addition of fresh, glucose-containing media significantly decreased AMPK phosphorylation (repeated measures ANOVA; time: p<0.01; time*treatment: 0.73; treatment: p = 0.082). B) Fresh media addition increased GSK3β phosphorylation (repeated measures ANOVA; time: p<0.001; time*treatment: p = 0.262; treatment: p = 0.424), although not significantly different between groups treated with either 0.5 or 5.5 mM glucose. Shown is densitometric analysis of the ratio of phosphorylated over total protein. Phosphorylation at 0 h was set to 100%. Note the different time points between pAMPK/AMPK and pGSK/GSK. Plotted are mean values ± SEM of 4 independent experiments.

After a 12 h serum starve in 0.5 mM glucose, media was replaced with fresh media containing either 0.5 or 5.5 mM glucose, following which phosphorylation levels of AMPK and GSK3β were assessed for 12 h or 4 h, respectively, using Western blot analysis. Serum starvation resulted in high levels of phosphorylated AMPK, whereas addition of fresh media significantly decreased levels of pAMPK (repeated measures ANOVA; time: p<0.01; time*treatment: p = 0.73; treatment: p = 0.082) (Fig 2A). AMPK phosphorylation was at least 50% repressed during the 12 h incubation with 5.5 mM glucose, whereas there was a trend that the phosphorylation levels of AMPK were reduced to a lesser extent in the 0.5 mM glucose group (Fig 2A), but this did not reach statistical significance. Serum starvation resulted in low levels of GSK phosphorylation. Phosphorylation significantly increased upon replacement with fresh media, although no significant differences were detected between cells treated with either 0.5 or 5.5 mM glucose (repeated measures ANOVA; time: p<0.001; time*treatment: p = 0.262; treatment: p = 0.424) (Fig 2B).

### 12.5 μM of Compound C inhibits AMPK phosphorylation and Per2 and Bmal1 transcription

In order to test the hypothesis that the effect of glucose on the rhythmic expression pattern of *Per2* was through decreased activity of AMPK, which was based on the trend towards a difference between the two glucose concentrations as seen in Fig 2A, phosphorylation (activity) of AMPK was chemically inhibited using Compound C (CC; dorsomorphin). Further repression of AMPK and corresponding alterations in the rhythmic expression pattern of *Per2* mRNA levels would indicate that AMPK is involved in the glucose-mediated effects on the clock.

**Fig 3:**
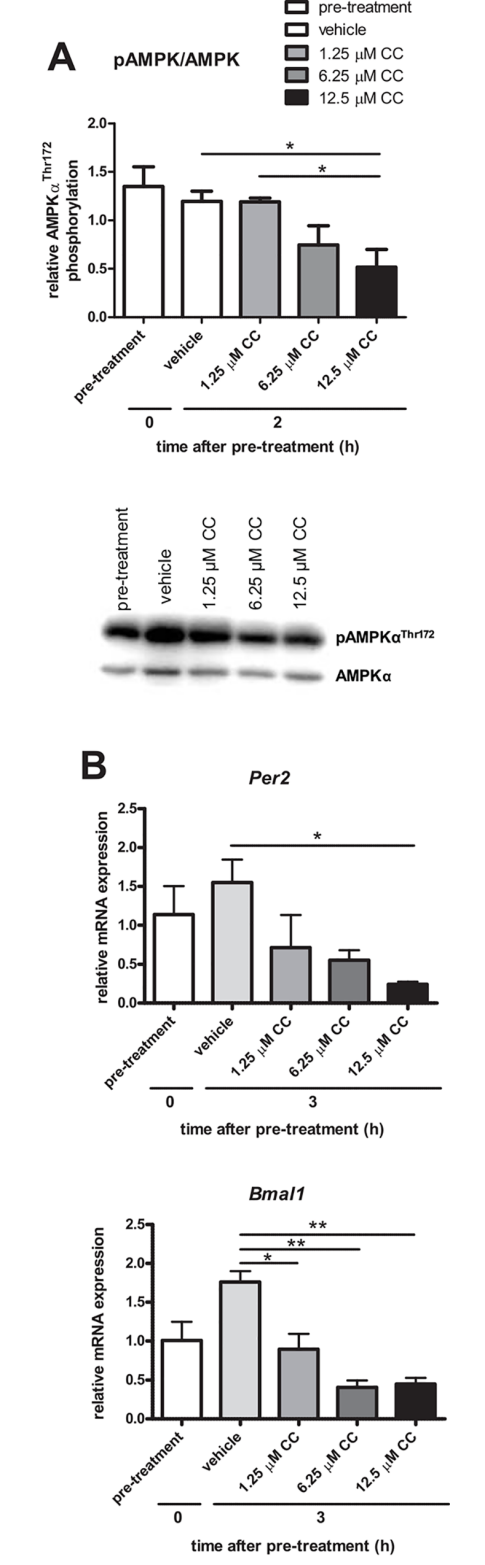
Compound C inhibits AMPK phosphorylation status and has a dose-dependent effect on *Per2 and Bmal1* transcriptional expression. After serum starvation, cells were pre-treated with 25 μM Compound C for 1 h, followed by media replacement containing either 5.5 mM glucose supplemented with vehicle (water), or Compound C in the following final concentrations: 1.25, 6.25 or 12.5 μM. Protein and RNA were harvested after 2 and 3 hours, respectively. A) AMPK phosphorylation status was determined using Western blot analysis. Shown is densitometric analysis of the ratio of phosphorylated over total protein. Mean ± SEM of 3 independent experiments, with a representative Western blot. B) Relative mRNA transcript levels of *Per2* and *Bmal1*, normalized to *Histone 3a* mRNA levels. Graphs display mean ± SEM of 3 independent experiments. *:p<0.05; **:p<0.01 as determined by one-way ANOVA followed by Tukey’s post-hoc test.

Efficacy of Compound C in the inhibition of AMPK phosphorylation and transcriptional expression of *Per2 and Bmal1* was tested using increasing doses of Compound C at 2 h or 3 h after media replacement, as indicated. Compound C shows a dose-dependent inhibitory effect on AMPK phosphorylation, with a significant repression using 12.5 μM, and no repression using 1.25 μM of Compound C (Fig 3A). Transcriptional expression of *Bmal1* shows a dose-dependent repression, which is significant starting from 1.25 μM Compound C (Fig 3B). *Per2* expression was significantly repressed using 12.5 μM Compound C (Fig 3B).

### Compound C inhibits Per2 and Bmal1 transcription, independent of AMPK phosphorylation

As 12.5 μM Compound C was able to repress both AMPK phosphorylation and *Per2* and *Bmal1* transcription, this concentration was further used to assess the effects of AMPK inhibition on the rhythmic expression profile of *Per2* and *Bmal1*. Cells were incubated with serum containing either 0.5 mM or 5.5 mM glucose in the absence or presence of 12.5 μM Compound C. AMPK phosphorylation and transcriptional expression of *Per2 and Bmal1* were assessed over 12 h following media change.

**Fig 4:**
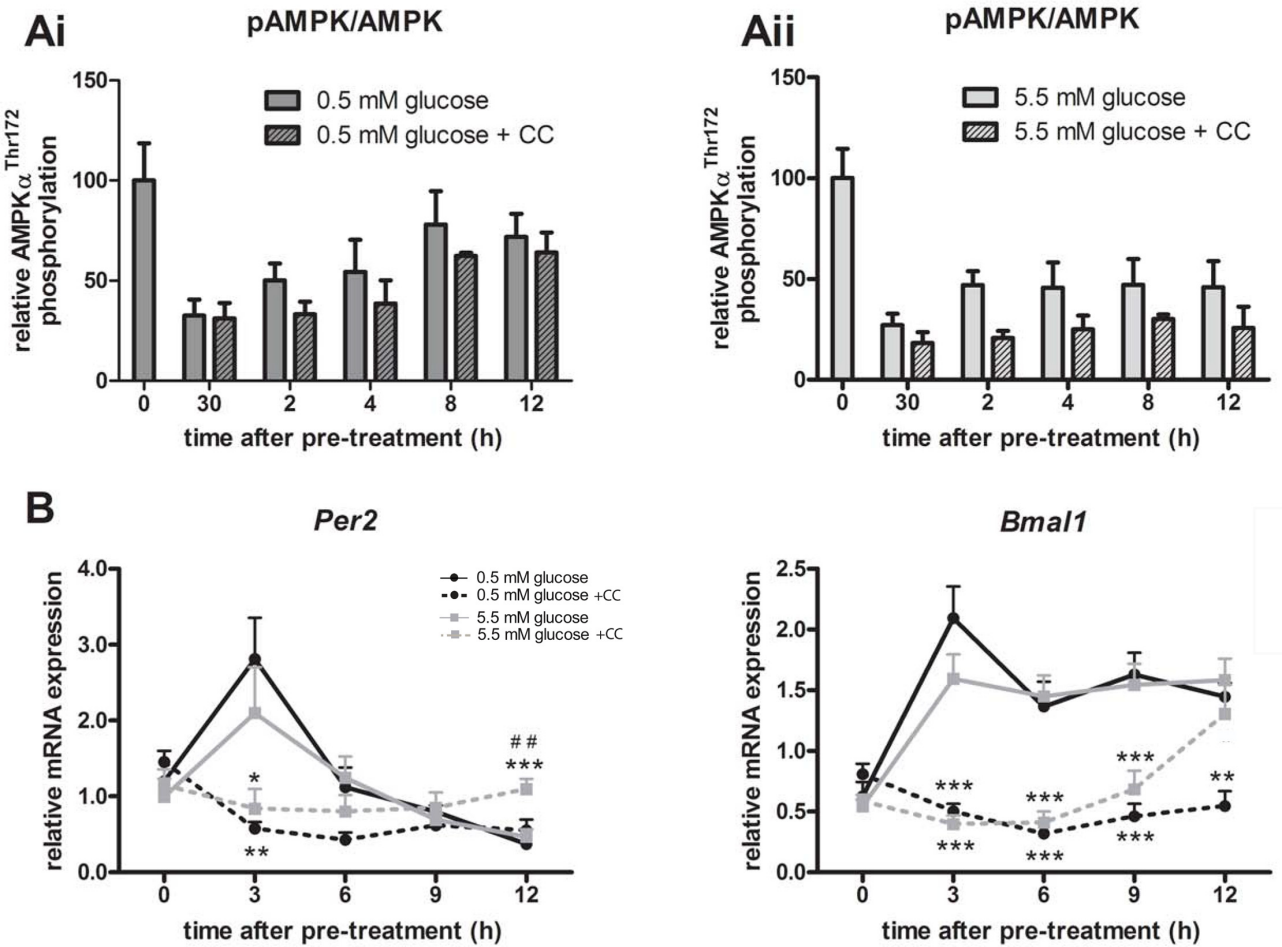
Effects of Compound C on phosphorylation status of AMPK and the transcriptional expression profile of *Per2* and *Bmal1*. After serum starvation, cells were pre-treated with 25 μM Compound C (CC), followed by replacement of the media with either 0.5 or 5.5 mM glucose in the absence or presence of 12.5 μM CC. This was considered time 0. Protein and RNA was harvested at indicated time points. Ai) Relative levels of AMPK phosphorylation in mHypoE-37 neuronal cells in 0.5 mM and Aii) 5.5 mM glucose-containing media in the absence or presence of 12.5 μM of the AMPK inhibitor Compound C (CC). Shown is the densitometric analysis of the ratio of phosphorylated over total protein. B) Treatment with 12.5 μM Compound C significantly alters *Per2 and Bmal1* transcriptional expression profile in mHypoE-37 neuronal cells. Cells were harvested every three hours during 12 hours and transcriptional expression was measured using qRT-PCR. Compound C inhibited *Per2* expression at 3 hours after media replacement, and increased *Per2* expression at 12 h after media replacement. The transcriptional expression of *Bmal1* was suppressed by CC at every time point *: P<0.05; **: P<0.01; ***: P<0.001 between glucose with and without CC. ##: p<0.01 between 0.5 mM glucose + CC and 5.5 mM glucose + CC. Determined by repeated measures ANOVA, followed by one-way ANOVA for each individual time point with a Tukey’s post-hoc test. All values are relative to mRNA levels of *Histone 3a*. Mean ± SEM of 4 independent experiments.

12.5 μM of Compound C decreased phosphorylation of AMPK after media change in the 0.5 mM glucose group (Fig 4Ai), and in the 5.5 mM glucose group (Fig 4Aii). However, although there appeared to be a trend towards a decrease in pAMPK with CC with both 0.5 and 5.5 mM glucose, the results did not reach statistical significance. In the same experiment, mRNA levels of *Per2* and *Bmal1* were analyzed after treatment with 0.5 mM or 5.5 mM glucose, in the presence or absence of 12.5 μM of Compound C. Transcript levels of *Per2* were significantly suppressed by Compound C at 3 h after pre-treatment, whereas at 12 h, *Per2* expression was higher in the 5.5 mM glucose with Compound C as compared to vehicle (Fig 4B). Transcription of *Bmal1* was significantly repressed at every time point after pre-treatment in the 0.5 mM group with Compound C as compared to vehicle (Fig 4B). In the 5.5 mM group, the repression of *Bmal1* was significant at 3, 6, and 9 h after pre-treatment (Fig 4B). The initial peak of *Per2* and *Bmal1* at 3 h was completely abolished by Compound C, resulting in the loss of a rhythmic expression pattern for these genes in the presence of Compound C.

**Fig 5:**
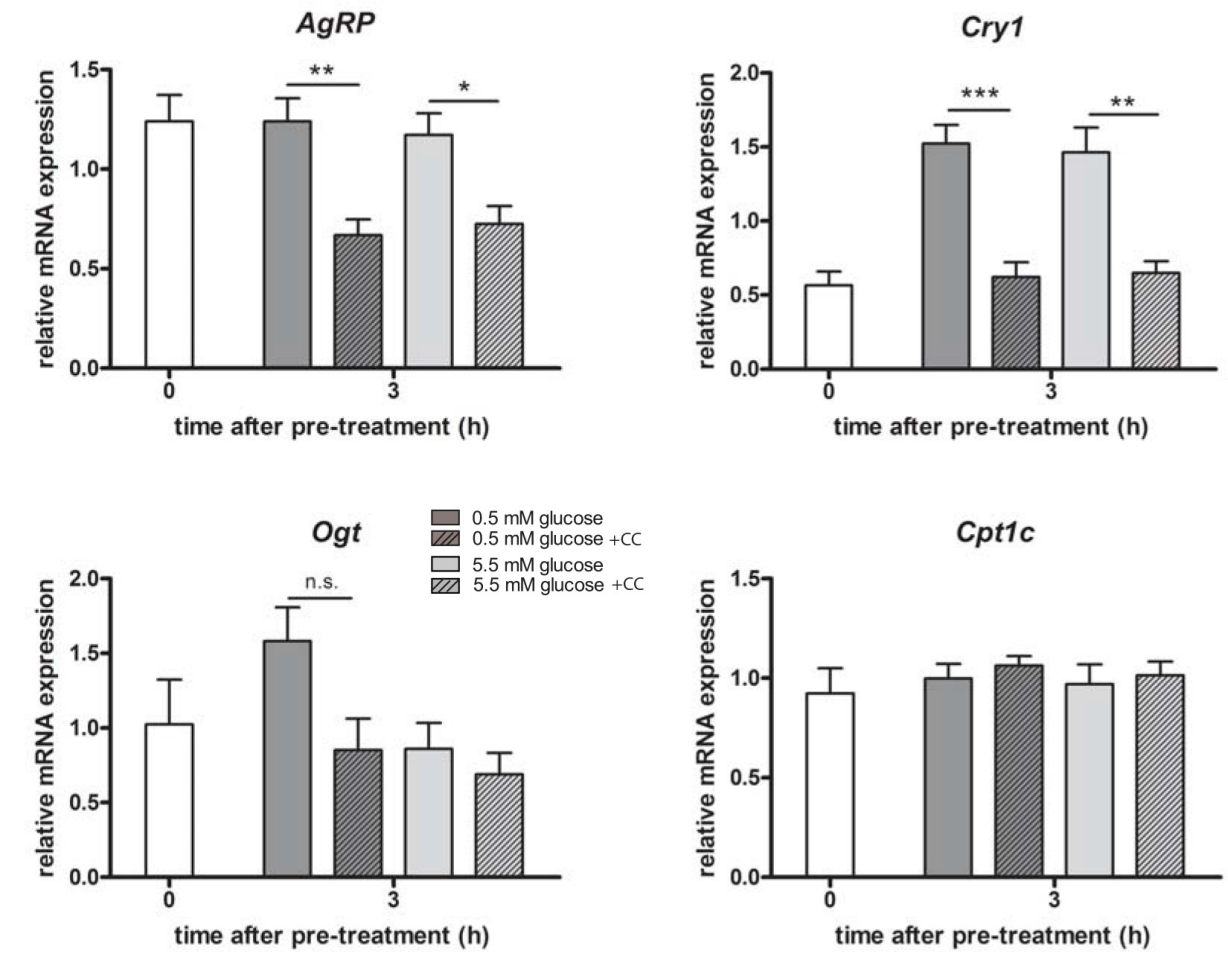
Compound C significantly inhibits transcriptional expression of *AgRP* and *Cry1*, but not *Cpt1c* and *Ogt*. After serum starvation, mHypoE-37 cells were pre-treated with either vehicle or 25 μM Compound C, followed by replacement of the media with either 0.5 or 5.5 mM glucose in the absence or presence of 12.5 μM CC. This was considered time 0. RNA was harvested at indicated time points. Shown are mRNA levels relative to *Histone3a* mRNA levels. Plotted are mean values ± SEM of 4 independent experiments. *: p<0.05; **:p<0.01 as determined by one-way ANOVA followed by Tukey’s post-hoc test. N.s.: not significant.

Compound C is a potent, selective and reversible AMPK inhibitor, but it has been reported that, in cancer cells, it suppresses transcriptional expression in a number of genes, independently of AMPK [40]. To test if the repression of *Per2 and Bmal1* mRNA levels in the mHypoE-37 hypothalamic neurons was the result of general transcriptional repression by Compound C, mRNA expression of agouti-related peptide (*AgRP)*, Cryptochrome1 *(Cry1)*, carnitine palmitoyltransferase 1C (*Cpt1c)*, and O-linked N-acetylglucosamine transferase (*Ogt)* were analyzed. *AgRP*, *Cry1* and *Cpt1c* have been reported to be regulated by AMPK [39, 41], whereas no relationship has been reported between AMPK and *Ogt*. *AgRP* and *Cry1* mRNA levels were significantly repressed by Compound C, both in the 0.5 and 5.5 mM glucose groups (Fig 5). *Ogt* transcript levels trended to be reduced (p = 0.067) in the 0.5 mM group treated with Compound C, whereas no differences were detected in the 5.5 mM group treated with or without CC (Fig 5). *Cpt1c* mRNA levels were unaffected by CC treatment (Fig 5). Furthermore, transcript levels of the normalization gene *histone3a* were not affected by Compound C treatment (data not shown).

## Discussion

The circadian clock and metabolism display an intricate relationship and it has been shown that alterations in normal clock function can result in alterations in metabolism. The use of cell lines to study the regulation of circadian rhythms allows for a thorough examination of the direct effects of nutrients on specific hypothalamic neuronal populations. To assess whether different concentrations of glucose can affect rhythmic expression of core clock genes in the hypothalamus, we used the non-SCN, hypothalamic cell line mHypoE-37, which was previously shown to express the core circadian genes, and that the transcript levels of *Bmal1*, *Per2*, *Rev-erbα*, and *Cry1* endogenously cycle with a circadian period [19].

Nutrients can affect the peripheral molecular clock (reviewed in [16]). Glucose is an important signal to reset the clock, and an inducer of circadian rhythms *in vitro*, in a peripheral cell model [20]. Alterations in the temporal availability of glucose may possibly lead to disruptions in peripheral clock gene expression, desynchronization of peripheral clocks from SCN control, or desynchronization between clock genes and metabolic genes, which may eventually contribute to metabolic abnormalities.

To identify the effect of glucose on the circadian expression profile of two core clock genes, mHypoE-37 neuronal cells were subjected to media containing low (0.5 mM) or high (5.5 mM) glucose, following which transcriptional expression of *Per2 and Bmal1* was assessed over a period of 36 hours. *Per2 and Bmal1* were chosen, as they represent core clock genes of the opposing arms of the molecular clock translational-transcriptional feedback loop. Extracellular glucose concentrations in the rat brain are approximately 20% of serum glucose levels and range from ~0.2 mM during systemic hypoglycemia to ~4.5 mM during systemic hyperglycemia [42]. Treatment of the mHypoE-37 neurons with 0.5 and 5.5 mM glucose therefore represents a state of hypo- and hyperglycemia, respectively. In the whole organism, alterations in glucose levels are rapidly counter-regulated to restore normoglycemia. In this isolated hypothalamic cell line, there is no counter regulation from the periphery nor activation by the autonomic nervous system to alter hepatic glucose production, and therefore this cell model enables to study the isolated effects of glucose on the molecular clock in non-SCN hypothalamic cells.

The rhythmic expression profile of *Bmal1* mRNA was not affected by different concentrations of glucose, whereas high glucose concentrations shortened the period, and caused a phase-delay in the expression pattern of *Per2*. The findings for *Per2* are in line with Hirota *et al*., who reported that the induction of a circadian rhythm in rat fibroblasts by glucose was preceded by a rapid down-regulation of *Per1* and *Per2* [20]. The results for *Bmal1* are in contrast to a study by Lamia *et al*., who reported that higher concentrations of glucose resulted in a decrease of *Bmal1* period and increase in *Bmal1* amplitude in U2OS cells (human bone osteosarcoma cells), dependent on AMPK levels. It is quite possible that glucose exerts differential effects on human/peripheral/bone/cancer and hypothalamic cells [31].

As nutrient sensors have intricate links to the molecular clock, we assessed whether GSK3β and AMPK activity were affected by different concentrations of glucose in the media. The addition of fresh glucose-containing media after a 12 h serum starve in low-glucose media, increased and decreased phosphorylation status of GSK3β and AMPK, respectively, indicating an inactivation of GSK3β and AMPK [43]. This is in line with the literature and demonstrates that the mHypoE-37 neurons are able to sense glucose. Interestingly, there was no difference in GSK3β phosphorylation between the high and low concentration of glucose. AMPK phosphorylation was consistently lower in the high glucose group, but this trend did not reach statistical significance at any time point. This could be due to the level of variance between the groups. We speculate that the levels of glucose in the medium were able to maintain basal levels of AMPK activity, and only in the absence of glucose would we see a dramatic change in AMPK levels, as we found levels of phospho-AMPK to be significantly elevated in medium without glucose (data not shown; DDB unpublished observation). Because we did not think that 0 mM glucose was representative of any physiological situation, we decided to change our experimental paradigm to include 0.5 mM glucose as our low glucose measure, but lower levels may have shown significant activation of AMPK in the mHypoE-37 neurons depending upon their sensitivity to glucose.

Despite the fact that there was little difference in AMPK phosphorylation between the glucose concentrations, we hypothesized that the effects of glucose on the molecular clock could be through differential activation of AMPK as a general energy sensor. In order to assess whether inhibiting AMPK activation could lead to a subsequent decrease in *Per2* mRNA expression, AMPK was chemically inhibited using Compound C. Compound C did repress AMPK activity over a period of 12 h with the largest repression seen in the 5.5 mM glucose group. Although this repression was not statistically significant, it does indicate that Compound C was able to inhibit AMPK phosphorylation. In the same experiment, it was shown that Compound C dramatically inhibited *Per2* and *Bmal1* mRNA expression over a 12 h time period, to the point where no rhythms were detected. The effects of Compound C on *Bmal1* were unexpected, as *Bmal1* was not affected by different concentrations of glucose and subsequent alterations in AMPK phosphorylation status.

The initial peak in expression of *Per2* and *Bmal1* at 3 h after synchronization was abolished after treatment with Compound C, leading to a complete loss of rhythmic expression of those genes in the first 12 h (Fig 4B). Interestingly, this impressively significant repression was not observed with AMPK phosphorylation. Similarly, using different doses of Compound C (Fig 3), it was shown that AMPK phosphorylation was not yet affected, while *Per2 and Bmal1* were already inhibited. This could indicate that the effect of Compound C on *Per2 and Bmal1* was likely independent of AMPK. AMPK-independent effects of Compound C on gene expression in the neurons were further exemplified in Fig 5. *AgRP*, *Cpt1c* and *Cry1* gene expression are all known to be dependent on AMPK phosphorylation. The results with *Ogt* are potentially interesting, as it appears that Compound C may differentially repress *Ogt* at 5.5 mM, but not 0.5 mM (p = 0.067), although this did not yet reach statistical significance. *Ogt* has been linked to brain glucose metabolism, as well as insulin resistance [44, 45], and may have a unique role in the hypothalamus at different glucose concentrations. Moreover, *Ogt* levels and activity are increased in AgRP neurons during fasting [46], as is corroborated by our results in the mHypoE-37 neurons. Further studies will be performed to determine the significance of CC-mediated repression at 0.5 mM glucose, but not 5.5 mM glucose. Despite the inhibition of AMPK phosphorylation, *Cpt1c* was unaffected by Compound C, again indicating an AMPK-independent effect of Compound C, as well as specificity of the Compound C effects in the neurons.

Although Compound C is the only available, and most widely used, cell-permeable AMPK inhibitor, it also exerts AMPK-independent biological effects, including inhibition of bone morphogenetic protein signaling [47]. It has also been shown to inhibit a number of kinases other than AMPK [48]. Furthermore, Compound C has been reported to prevent hypoxia inducible factor-1 activation in mouse embryonic fibroblasts, and inhibit the proliferation and differentiation of preadipocytes by stabilizing and inducing P21 levels, independent of AMPK inhibition [49]. To our knowledge, the effects of Compound C on the molecular clock have not been reported. These findings are of significance to many metabolic processes dependent upon the molecular clock, and could have major ramifications on overall physiology.

In cancer cells, it has been reported that Compound C suppresses transcriptional expression of a number of genes, independently of AMPK [40]. Therefore, it could be speculated that the inhibitory effects on the transcriptional profile of *Per2 and Bmal1* are the result of ubiquitous transcriptional repression, rather than an effect of Compound C on these genes specifically. However, in this study we found that not all of the genes studied were equally affected by Compound C (Fig 5), indicating a specific effect on the clock genes, and not a general transcriptional repression, although an underlying mechanism needs to be elucidated. Furthermore, studies reporting the anti-proliferative effects of Compound C have all been performed in cancer cell lines. For instance, Compound C inhibited glioma proliferation in glioma cells through multiple mechanisms independent of AMPK, including inhibition of Akt and mTORC1/C2, cell-cycle block at G2-M, and induction of necroptosis and autophagy, whereas normal astrocytes were significantly less susceptible to Compound C [50].

We therefore demonstrate for the first time the direct effects of Compound C on clock gene expression, as well as AgRP mRNA levels, in hypothalamic neurons. Indeed, previous studies have reported AMPK-independent effects of Compound C, but few studies used non-cancer cell lines. Compound C completely abolished the circadian expression profile of *Per2 and Bmal1*, which may affect many clock-controlled genes, especially when taking into account that 8–10% of the entire transcriptome is under clock control.

In conclusion, we have shown that the circadian expression profile of *Per2* in the non-SCN, hypothalamic neuronal cell line mHypoE-37 was affected by glucose, whereas the circadian expression profile of *Bmal1* was unaffected. As we did not found significant effects of these glucose concentrations on AMPK phosphorylation, we suggest that the effects are AMPK-independent. Chemical inhibition of AMPK phosphorylation did not result in significant downregulation of AMPK phosphorylation, while at the same time *Per2*, *Bmal1*, *Cry1*, and *AgRP* mRNA expression levels were dramatically repressed, indicating an AMPK-independent effect of Compound C on these clock genes. Therefore, we suggest that caution should be taken in interpreting results on the circadian clock, or downstream metabolic genes or physiological responses when Compound C is used as an AMPK inhibitor.
